# Neuroanatomy Learning: Augmented Reality vs. Cross‐Sections

**DOI:** 10.1002/ase.1912

**Published:** 2019-07-19

**Authors:** Dylan J.H.A. Henssen, Loes van den Heuvel, Guido De Jong, Marc A.T.M. Vorstenbosch, Anne‐Marie van Cappellen van Walsum, Marianne M. Van den Hurk, Jan G.M. Kooloos, Ronald H.M.A. Bartels

**Affiliations:** ^1^ Department of Anatomy Radboud University Medical Center Nijmegen The Netherlands; ^2^ Donders Institute for Brain, Cognition and Behavior Radboud University Medical Center Nijmegen The Netherlands; ^3^ Department of Neurosurgery Radboud University Medical Center Nijmegen The Netherlands; ^4^ Department of Educational Sciences, Faculty of Social Sciences Radboud University Nijmegen The Netherlands

**Keywords:** neuroanatomy education, medical education, undergraduate education, augmented reality, cross‐sectional anatomy, neuroanatomy, neuroscience

## Abstract

Neuroanatomy education is a challenging field which could benefit from modern innovations, such as augmented reality (AR) applications. This study investigates the differences on test scores, cognitive load, and motivation after neuroanatomy learning using AR applications or using cross‐sections of the brain. Prior to two practical assignments, a pretest (extended matching questions, double‐choice questions and a test on cross‐sectional anatomy) and a mental rotation test (MRT) were completed. Sex and MRT scores were used to stratify students over the two groups. The two practical assignments were designed to study (1) general brain anatomy and (2) subcortical structures. Subsequently, participants completed a posttest similar to the pretest and a motivational questionnaire. Finally, a focus group interview was conducted to appraise participants’ perceptions. Medical and biomedical students (*n* = 31); 19 males (61.3%) and 12 females (38.7%), mean age 19.2 ± 1.7 years participated in this experiment. Students who worked with cross‐sections (*n* = 16) showed significantly more improvement on test scores than students who worked with GreyMapp‐AR (*P* = 0.035) (*n* = 15). Further analysis showed that this difference was primarily caused by significant improvement on the cross‐sectional questions. Students in the cross‐section group, moreover, experienced a significantly higher germane (*P* = 0.009) and extraneous cognitive load (*P* = 0.016) than students in the GreyMapp‐AR group. No significant differences were found in motivational scores. To conclude, this study suggests that AR applications can play a role in future anatomy education as an add‐on educational tool, especially in learning three‐dimensional relations of anatomical structures.

## INTRODUCTION

It is important for clinicians to understand human neuroanatomy as it supports the establishment of a diagnosis (Frank and Danoff, 2007). While it is important, neuroanatomy is also perceived to be challenging, requiring thorough basic knowledge, insight and practice with insightful preparations, clear atlases and properly designed ICT applications (Fitzgerald et al., 2008). Traditionally, neuroanatomy education has been carried out using specimens (Albanese, 2010), but growing numbers of students, changing curricula (Frenk et al., 2010) and less time being assigned to anatomy subjects in curricula (Drake et al., 2009; Louw et al., 2009; Bergman et al., 2011) have urged the exploration of new teaching methods. The development and use of alternative methods for teaching neuroanatomy, therefore, are expected to increase in the next few years (Shaffer, 2004; Winkelmann, 2007). Alternative teaching methods in the form of technological innovations will appeal to the modern, digital student (Pani et al., 2013; Drapkin et al., 2015; Allen et al., 2016; Arantes et al., 2018). Various reports have shown that students have a preference for new teaching methods because they are considered to be interactive, engaging, and widely available (Shen et al., 2008; Huang et al., 2010; Drapkin et al., 2015).

One of the proposed new teaching methods is three‐dimensional (3D) visualization technology, such as augmented reality (AR). As AR offers the possibility to augment the real world with virtual sensory information such as sound, touch, or images (Ma et al., 2016), it can offer a highly realistic situated learning experience that is supportive to complex medical learning (Ma et al., 2016). An important advantage of AR over physical models and cross‐sections in learning anatomy is that it offers the opportunity to thoroughly get to know the anatomy of a structure by virtually disassembling parts and putting them back together. A possible disadvantage of AR is the absence of tactile feedback (Kamphuis et al., 2014).

In 2015, a meta‐analysis of various anatomical teaching methods (i.e., 3D visualization techniques, dissection, cross‐sections, and two‐dimensional images) showed that 3D visualization technology yielded significantly better results with regard to the acquisition of spatial knowledge (Yammine and Violato, 2015). However, the same study also showed that, after learning anatomy with 3D visualization technology, actual knowledge did not always improve significantly (Yammine and Violato, 2015). Subgroup analysis showed that learning anatomy with 3D visualization technology only benefited junior students and when students were studying musculoskeletal anatomy. Other studies also show that technology‐based learning methods are not always superior to classic teaching methods (Garg et al., 1999, 2001, 2002; Brenton et al., 2007).

An explanation for this phenomenon might be found in the cognitive load theory. The cognitive load theory was initially developed in the 1980s and aimed to develop instructional design principles and strategies based on a model of human cognitive architecture (Sweller, 1988). Cognitive load theory assumes that the human cognitive system has a limited working memory which cannot contain more than five to nine novel elements (Ockelford, 2002). It also assumes that working memory actively processes no more than two to four new elements simultaneously and that almost all new information is lost after about 20 seconds unless it is rehearsed. The theory emphasizes that these rules only apply to novel information, obtained through sensory memory (Sweller, 1988; Van Merriënboer and Sweller, 2010).

Generally, cognitive load is divided into three categories: intrinsic, extraneous, and germane cognitive load. Intrinsic cognitive load is imposed by elements in the content information and is closely related to subject matter complexity. Extraneous cognitive load can be imposed by poorly designed instructional materials, which is ineffective and does not contribute to the construction and the automation of schemas in the brain. Germane load is created by effective instructional strategies that foster the process of schema construction and automation (Leppink, 2017). Schemas are procedures or ways of organizing knowledge or mental data (Khalil et al. 2005a,b). To increase the germane load, learners must engage in cognitive activities such as elaboration, abstraction, and drawing inferences (Sweller and Chandler, 1994). The sum of intrinsic, extraneous, and germane loads is equal to the total cognitive load, which, to be effective, should not exceed the memory resources available for learning (Sweller and Chandler, 1994; Paas et al., 2003; Cierniak et al., 2009). In general, it can be said that germane load needs to be maximized, intrinsic load should be optimized and extraneous load should be minimized for optimal learning (Khalil et al. 2005a,b).

The complexity of anatomical structures and their relationships account for high intrinsic cognitive load (Garg et al., 2002; Seixas‐Mikelus et al., 2010). It also has to be kept in mind that making visuals more dynamic and interactive does not always result in reduced extraneous and increased germane cognitive load (Sweller and Chandler, 1994). Dynamic visualizations have characteristics that are frequently overlooked when they are used for instructional purposes: effects of duration and displacement. Limited duration when presenting information to working memory may hinder the retention of temporary information if it is not subsequently used (Khalil et al., 2005a). This is in contrast with long‐term memory, which can store knowledge permanently and has an unlimited capacity. Displacement means that learning new material has a negative effect on the retention of previously learned materials (Khalil et al., 2005b). In an AR learning environment, however, students can also be cognitively overloaded by the amount of information they face (Wu et al., 2013), causing, for example, 3D models to be remembered as viewpoint‐specific two‐dimensional images (Bulthoff et al., 1995). Hence, students need to mentally rotate the AR projected or displayed anatomical structures to align them with the more traditional anatomical views in their textbooks (i.e., the sagittal, frontal, and axial views), creating additional extraneous cognitive load (DeLeeuw and Mayer, 2008; Van Merriënboer and Sweller, 2010; Khot et al., 2013). Using physical models or specimen brains to learn neuroanatomy has the advantage that students can rotate the brain physically, which has been suggested to reduce extraneous cognitive load (Khot et al., 2013; Preece et al., 2013). However, working with AR has also been suggested to possibly reduce cognitive load (Küçük et al., 2016). This can possibly be explained by findings in literature which report that when AR applications are well‐designed, better learning can be achieved with less cognitive load (Iordache et al., 2012; Di Serio et al., 2013). Such well‐designed AR application adheres to the spatial and the temporal continuity principles of the cognitive theory of multimedia learning (Mayer, 2009; Wilson, 2015). The principles state that learning from systems that present learning materials in a well‐integrated and organized application can avoid incidental cognitive load (Chiang et al., 2014).

In The Netherlands, basic sciences, including anatomy, are primarily taught in the first three years of the Medicine and Biomedical Sciences curriculum, a period also referred to as the Bachelor’s program. In this preclinical program, all students of medicine and biomedical sciences receive 15 hours of neuroanatomy education; 10 hours of individual assignments, 2 hours of lectures, and 3 hours in the dissection rooms (Radboud Health Academy, 2018). Anatomy courses mainly consist of small group assignments, interactive lectures and student‐centered practical assignments with the use of teacher‐written instructions in the dissection rooms. Prosected specimens, cross‐sections, and plastic models are provided for students to learn anatomy in the dissection rooms. Students are required to bring their own anatomy atlases (Bergman, 2014). Furthermore, white matter prosections are combined with tissue plastination to produce detailed white matter specimens that are durable and easy to handle and do not require special care or conditions (Arnts et al., 2014). Such plastinated specimens are available for students in the dissection rooms. In one of the sessions in the dissection rooms, furthermore, medical students can model the subcortical structures using colored clay (Kooloos et al., 2014).

**Figure 1:**
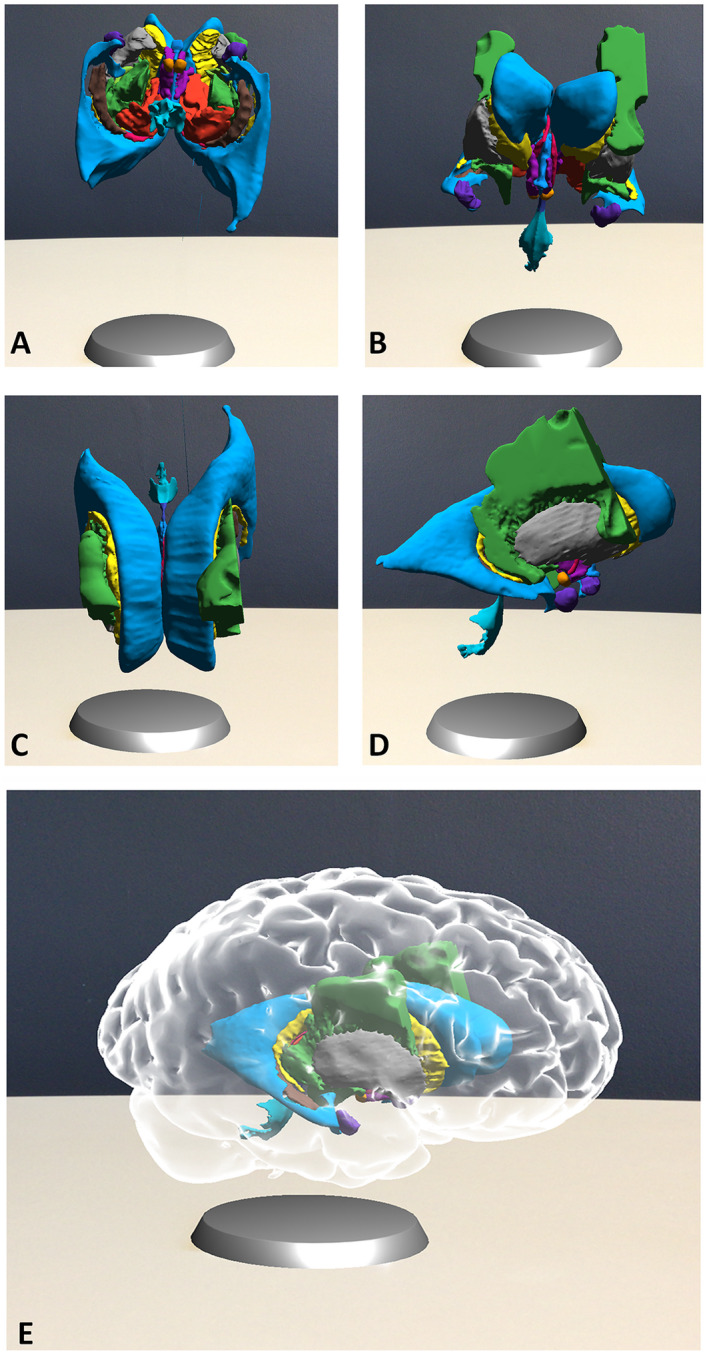
Exemplary images of the augmented reality feature of GreyMapp. The cortical outline has been made invisible in order to show the model of the ventricles, basal ganglia, limbic system and part of the internal capsule. A, inferior view; B, anterior view; C, superior view; D, right lateral view; E, anterolateral view of the entire model.

**Figure 2:**
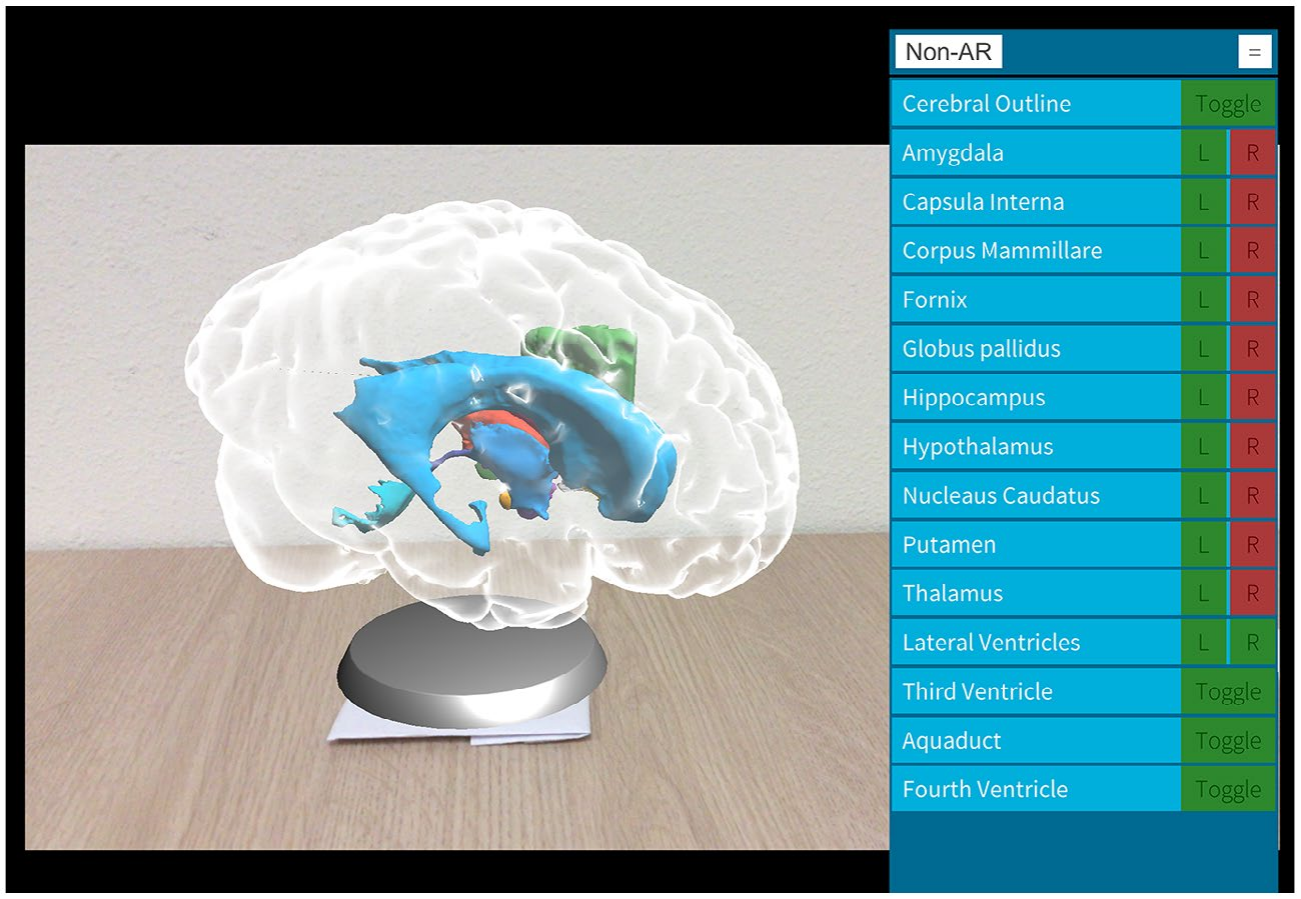
Capture from the GreyMapp‐AR application screen with a navigation panel.

**Figure 3:**
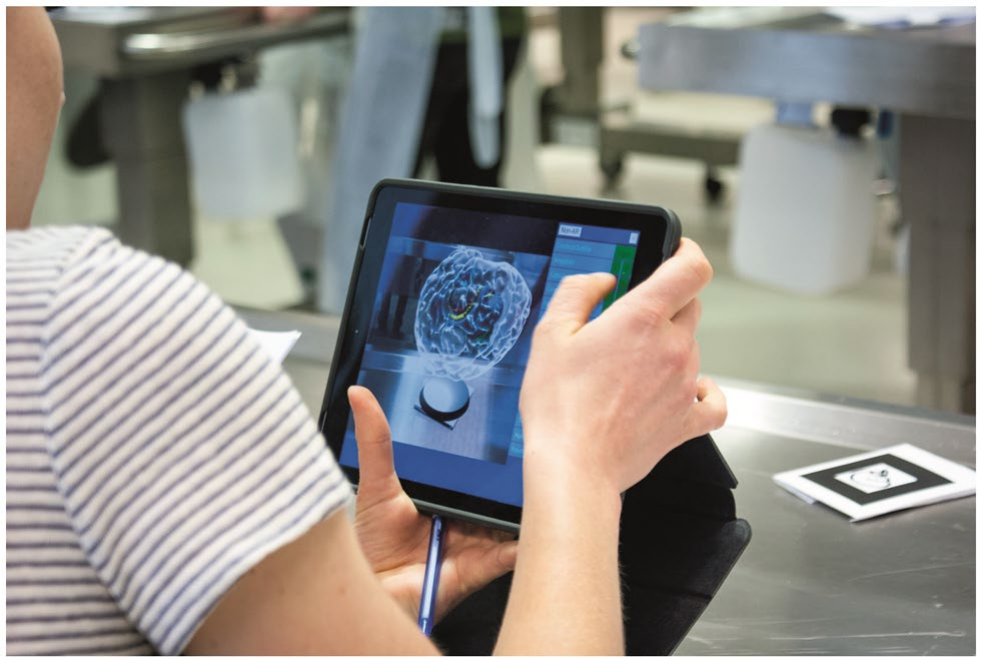
Student working with GreyMapp in the dissection room during the experiment.

In an attempt to optimize the learning effect during the scarce hours of neuroanatomy education, a new 3D visualization technology called GreyMapp was developed and first launched in 2016 (Henssen and De Jong, 2019). GreyMapp was designed with the aim of helping students master the 3D anatomy of the human brain in a modern and innovative way. GreyMapp allows users to navigate between an AR and a non‐AR environment, both containing the major structures of the basal ganglia, the limbic system, the internal capsule, and the ventricular system (Fig. 1). Figure 2 shows the GreyMapp‐AR screen with its navigation buttons (see also Supplementary Material Figure for more screen captures). Figure 3 shows the use of GreyMapp during a practical assignment in an educational setting in the dissection room. For this study, a tablet‐based version of the AR feature of GreyMapp (GreyMapp‐AR) was used. In the medicine and biomedical sciences curricula, knowledge of anatomy is assessed in a written theoretical examination. No practical examination takes place.

Other 3D visualization technologies for neuroanatomy learning are available around the globe. One example is the *Cerefy Atlas of Cerebrovasculature*, derived from a 3‐Tesla (3T) and 7‐Tesla (7T) magnetic resonance imaging (MRI) angiography (Nowinski et al., 2005, 2009a,2009b). The effects of this highly detailed model on learning, however, have never been investigated. Furthermore, this model mainly focuses on 3D vascular anatomy, which lies beyond the scope of GreyMapp. Another example was given by Pani et al. (2013), who described a monitor‐based computer program visualizing an accurate 3D graphic computer model of 19 structures in the human brain, based on the Visible Human Project (Pani et al., 2013). In addition to this 3D model, they created sections through different parts of the computer model. Both the 3D model and the sections, however, were simplified and modeled, causing loss of detail. Furthermore, the described monitor‐based program only functioned as a non‐AR model.

More AR‐focused models have been described as well. One of these is MagicBook, described by Küçük et al. (2016), in which students used smartphones to watch 3D video animations, a 3D anatomical model, and several diagrams to study neuroanatomy. MagicBook was found to improve student learning by making less cognitive effort. Moreover, MagicBook might have provided better learning satisfaction and enabled students to structure their knowledge to complete the learning tasks (Küçük et al., 2016). The MagicBook models and videos, however, focused on spinal cord anatomy and not on subcortical structures. In 2016, Allen et al. reported on a 3D, non‐AR model of cortical and subcortical structures. The authors found that students appreciated working with such models and that their learning outcomes improved significantly after learning with this model (Allen et al., 2016). This model, however, does not use an AR environment.

The innovative aspect of GreyMapp is that it is based on the mobile augmented reality education (MARE) design framework, derived by conceptual framework analysis methods, based on grounded theory, qualitative methods, and multidisciplinary research approaches (Clark and Mayer 2016; Zhu et al., 2015). Following the MARE design framework, three layers provide the hierarchical structure for the content objects. The design started, firstly, with defining learning objectives and placing them in the outcome layer. The foundation layer, secondly, contains the learning theories on which the tool (i.e., GreyMapp) was based. In this case, the use of GreyMapp in anatomy education was based on various articles (Khalil et al., 2005a; Estevez et al., 2010; Seixas‐Mikelus et al., 2010; Lee, 2012; Wu et al., 2013; Drapkin et al., 2015; Wilson, 2015; Yammine and Violato, 2015; Allen et al., 2016; Küçük et al., 2016; Moro et al., 2017). The third layer consists of factors that help to achieve the outcome layer. These factors include the learning environment (i.e., the dissection rooms), the learning activities (i.e., combining lectures, practical assignments and homework assignments with the option of studying neuroanatomy using GreyMapp), the learners’ personal paradigm (i.e., the learners’ own personal style of learning) and the learning assets (i.e., combining atlases, prosections, plastinated specimens, plastic models, and GreyMapp) (Zhu et al., 2015).

By applying the MARE design framework to the design and implementation of GreyMapp, the creators aimed to develop a well‐fitted, suitable, and innovative neuroanatomy education tool. For this study, however, the learning assets were modified in order to compare studying neuroanatomy using sections with GreyMapp‐AR. Due to the implemented stratification protocol, it was chosen not to take the learners’ personal paradigm into consideration. Another interesting feature of GreyMapp concerns the combination of AR and non‐AR environments, although the non‐AR environment was not used in this study. The postmortem, high‐resolution source data on which GreyMapp is based, finally, are another unique feature of GreyMapp.

The present study aimed to determine the effectiveness of GreyMapp‐AR in comparison with using cross‐sections of the brain in neuroanatomy learning. The primary outcome measure of this study was the difference in learning outcomes between GreyMapp‐AR and cross‐sectional anatomy learning as measured by the difference between pre and posttest scores. The secondary outcome measure was the participants’ experienced cognitive load and level of motivation in both groups. It was hypothesized that GreyMapp‐AR, when well aligned with the principles of cognitive load theory, would have a positive impact on the participants learning the 3D relations of subcortical structures.

## MATERIALS AND METHODS

### Participants

This study was conducted at the Faculty of Medical Sciences (Radboud University Medical Center) at Radboud University in Nijmegen, The Netherlands. First‐year students of Medicine and Biomedical Sciences were recruited as they had no neuroanatomy education prior to this experiment. Students who had previously engaged in other, extracurricular, and educational neuroanatomy activities were excluded. Students who had previously been enrolled in another Bachelor’s or Master’s program were also excluded. Students participated voluntarily and were recruited by means of recruiting announcements, online advertisements, and posters. Students could sign up for the experiment by sending an e‐mail to one of the researchers, after which they received an information letter about the experiment. After inclusion, participants were invited for one evening (duration 3.5 hours) to take part in the experiment. Prior to the experiment, all participants provided written informed consent. After eight weeks, subjects were asked to participate in a focus group interview. During this focus group interview, the participants’ experiences and perceptions in either group were assessed qualitatively.

### GreyMapp and GreyMapp‐Augmented Reality Applications

GreyMapp (Henssen and De Jong, 2019) was based on a 7T MRI scan of the human brain. For this application, the brain of an 83‐year‐old female donor was acquired via the body donor program at the Department of Anatomy of the Radboud University Medical Center, Nijmegen, The Netherlands. Cause of death was cardiac arrest, and the body was embalmed by perfusion with formaldehyde via the femoral artery within 23 hours after death. The brain was extracted from the cranial cavity by craniotomy within 24 hours after perfusion. Inspection of the brain by two neuroanatomists (D.H. and A.M.v.C.v.W.) showed no gross pathological characteristics, and the brain was stored in 7.7% formaldehyde for six months. Prior to MRI scanning, the brain was soaked in phosphate‐buffered saline (PBS 0.1M, pH 7.4) for five days to restore possible decreases in T_2_‐relaxation rates (Shepherd et al., 2009). The specimen was then placed in a plastic container filled with Novec FC‐3283 Fluorinert electronics liquid (3 M™, St. Paul, MN) for 24 hours, which is a susceptibility‐matched, hydrogen‐free liquid to decrease the free air in the blood vessels.

All imaging was performed using a 28‐channel knee coil in one overnight session using a Siemens Magnetom Terra 7T MRI imaging system (Siemens Corp., Erlangen, Germany). Structural imaging was performed at a resolution of 0.25 mm isotropic. Scanning parameters were: TE of 3.79 ms, TR of 7.58 ms, and a flip angle (*α*) of 35°.

The postmortem specimen was acquired from the body donor program at the Department of Anatomy of the Radboud University Medical Centre, Nijmegen, The Netherlands. All body donors in this program signed a written informed consent during lifetime permitting the use of their body and parts for science and teaching. The body donor program of the Radboud University Medical Center was approved by the national medical ethical committee of The Netherlands and legislated under Dutch law. Furthermore, the current postmortem study protocol was carried out in accordance with the recommendations of the CMO (Commissie Mensgebonden Onderzoek) region Arnhem‐Nijmegen, The Netherlands.

All neuroanatomical structures of interest were manually segmented in the T1‐weighted series by a junior neuroanatomist (D.H.) using an ITK‐SNAP medical image segmentation tool, version 4.13.1 (University of Pennsylvania, Philadelphia, PA) (Yushkevich et al., 2006). All segmentations were later evaluated by a second neuroanatomist (A.M.v.C.v.W.). Three‐dimensional volumes were created using ITK‐SNAP software and exported as mesh‐files in order to construct a 3D application. The application was constructed by a third investigator (G.d.J.) using the Unity cross‐platform game engine, version 5.6.3 (Unity Technologies ApS, San Francisco, CA). Figure 1 provides exemplary images of GreyMapp‐AR. Participants could study the displayed brain by focusing the tablet’s camera on a printed marker and then rotate the brain and learn about the brain’s different functions by touching the screen.

### Study Design and Outcome Measures

**Figure 4:**
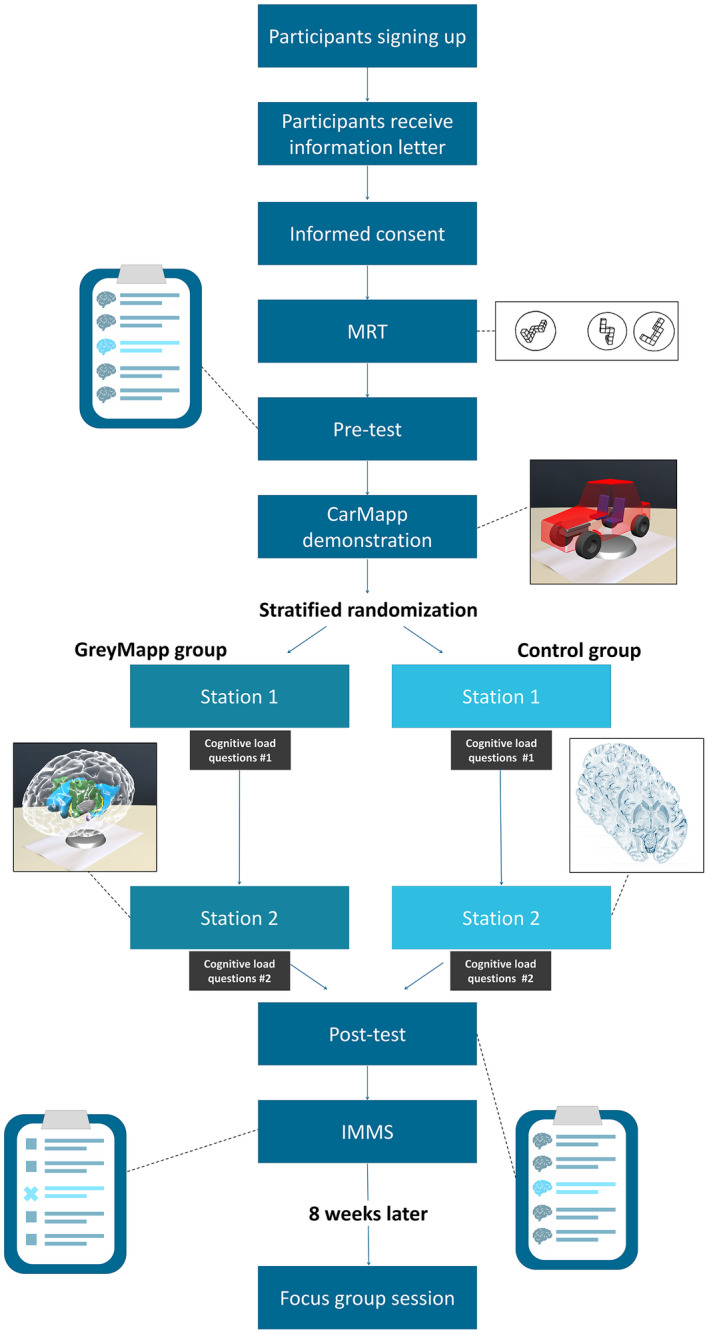
Overview of the study design. This study represents a two‐arm, multi‐staged design with stratified random sampling**.** IMMS, Instructional Measure of Motivation Survey; MRT, Mental Rotation Test.

This study had a two‐arm, multi‐staged design with stratified random sampling. Figure 4 depicts a flow chart of the study design. At the beginning of the experiment, all participants started with a mental rotation test (MRT) to measure spatial ability (Vandenberg and Kuse, 1978), (maximum score: 24 points). Then participants completed the pretest with neuroanatomy questions. The pre and posttests were used to measure knowledge of anatomy and were adapted from Kooloos et al. (2014). The format of the pre and posttests was identical, though they consisted of different variations of questions on spatial knowledge, covering identical content. The tests consisted of three parts: (1) a part containing extended matching questions (*n* = 9); (2) a part with multiple choice questions (*n* = 11); (3) a part with cross‐sections containing five outlined structures that needed to be named (Kooloos et al., 2014). Questions from parts 1 and 2 were always completed and handed in by the participants before the test containing part 3 was distributed. In this way, no cues from the illustrations in part 3 could be used to answer the questions in parts 1 and 2 (maximum score: 35 points (part 1: 9 points; part 2: 11 points; part 3: 15 points).

Based on Bloom’s taxonomy, the questions tested learning information belonging to the Application Dimension, in which learners are capable of implementing abstractions in particular and concrete situations that may be both similar and different from the learning situation (Bloom, 1956). An example of the test has been made available as Supplementary Material File 1. All three questions assessed 3D knowledge of anatomy, thus representing the complexity of the domain of 3D anatomy. All outcomes of the pre and posttests have been provided in percentages to facilitate comparability with other studies in this field.

After the pretest, participants were given a short introductory training by one of the researchers (G.d.J.) on working with an AR application similar to GreyMapp‐AR. This application, called CarMapp, had the same functions as GreyMapp‐AR but used a 3D car model in order to prevent transfer of learning of neuroanatomy knowledge. After the plenary training, all participants were able to work with CarMapp individually or in small groups. Subsequently, participants had a 40‐minute break, during which the researchers corrected the MRT forms. Based on the MRT results and the participants’ sex, stratified randomization took place. Participants were divided into two groups: (1) the GreyMapp‐AR group and (2) the control group, working with cross‐sections. Participants in the two stratified groups then performed two practical assignments in the dissection rooms. The instructions for the first practical assignment were completely identical for all participants. The first practical assignment provided a global overview of the anatomy of the human brain. In the second practical assignment, participants studied the subcortical structures of the brain in greater depth either using GreyMapp‐AR or using anatomical drawings of transverse sections of the human brain. The two groups were visually separated from each other by poster boards during this second practical assignment. Much like the normal situation, two senior anatomists (J.K. and M.V.) were present during these practical assignments to answer organizational and basic questions. No differences in question frequency or content were noticed between questions asked by participants in the GreyMapp‐AR or those in the control group.

After each of the two practical assignments, the participants were asked to answer three questions to measure the three dimensions of cognitive load. Cognitive load was measured by the same questions as described by Cierniak et al. (2009), see Supplementary Material File 2. The participants had to answer these questions on a six‐point Likert‐scale where 1 = not at all, 2 = just a little, 3 = somewhat, 4 = pretty much, 5 = very, and 6 = extremely (maximum score: 18 points; 6 points for each questions), (see Supplementary Material File 2).

Following the session at the dissection rooms, the students filled in the posttest and the Instructional Measure of Motivation Survey (IMMS). This survey was designed to measure the students’ motivational reactions to self‐directed instructional materials (Keller, 1987, 2010). The used version of the IMMS has been made available as Supplementary Material File 3.

### Explorations of Students’ Opinions about the Effects of GreyMapp‐Augmented Reality Application and on Utilization of Cross‐sections in Neuroanatomy Learning

When GreyMapp was designed, nine second‐year medical students, who were not included in this study, had the opportunity to work with GreyMapp several times to uncover possible errors in its programming and design. Following these practical assessments, nine individual interviews were held. The findings of these interviews were used to generate the grounded theory that the design of the AR application (i.e., GreyMapp‐AR) is of crucial importance in controlling cognitive load. This theory was supported by various articles reporting that students who worked with AR reported less cognitive load when attention was paid to the design of the AR applications (Iordache et al., 2012; Di Serio et al., 2013; Allen et al., 2016; Küçük et al., 2016).

The interviews, the literature and the results from the experiment described here together provided the framework for formulating the questions that were asked during the focus group interview. Pre‐defined questions included: “*How did you experience the experiment?*”; “*What part of this experiment was most educational and why?*”; “*What is your opinion of using technologies in modern anatomy education?*”; “*What advantages or disadvantages do you recognize when working with GreyMapp‐AR/cross‐sections?*.”

Eight weeks after the practical part of the experiment, participants were invited to participate in a focus group interview at the Radboud University Faculty of Medical Sciences. Results from the first part of the experiment were not communicated to the participants prior to the focus group interview, and open‐ended questions were used in the semi‐structured focus group interview to appraise perceptions of neuroanatomy learning using GreyMapp‐AR or using transverse sections. The focus group interview was audio recorded and moderated by an independent researcher (T.K.). One of the investigators (L.v.d.H.) was present during the interview and took additional notes. The focus group interview was conducted in an informal tone to increase all participants’ input and to encourage discussion of perspectives and thoughts. All participants were prompted to participate. An inductive iterative process was performed during the focus group interview using the constant comparative method, indicating that the interview could be steered in a different direction when a new topic arose. The moderator assessed when discussion of a topic had reached saturation. The total focus group interview lasted 60 minutes.

### Statistical Analysis

The statistical package SPSS Statistics, version 22 (IBM Corp., Armonk, NY) was used for statistical analyses. The Shapiro–Wilk test was applied to test normality of the acquired data. Descriptive statistical analyses were represented as mean with ± standard deviation (±SD) if normally distributed, or as a median with range (minimum‐maximum) if not normally distributed. Paired and unpaired student’s t‐tests were applied to compare mean scores between the (1) pre and posttests; (2) MRT scores, (3) cognitive load questions and (4) motivational questionnaires between the GreyMapp group and the control group.

Cronbach’s alpha test was used to assess the internal consistency (i.e., validity and reliability) of the pre and posttest scores. Internal consistency is generally regarded acceptable when ≥ 0.7. Kendall’s Tau‐b values were used to assess the association between experienced cognitive load and posttest score. To analyze whether the GreyMapp‐AR group and the control group had different distributions of categorical parameters (i.e., sex), a chi‐squared test was conducted. A repeated measures ANCOVA was conducted for the numerical data and investigated two factors that could have a possible interaction effect on the experiment. These two factors were (1) Time and (2) Group (GreyMapp‐AR vs. transverse, cross‐sections). A repeated measures ANCOVA was chosen because the to‐be‐investigated groups were dependent, the measures (pre and posttest) were equal and because it is known to control for covariates.

The control group worked with cross‐sections in their second practical assignment. This was considered to be a probable confounder of the results of this experiment, even though the anatomical sections in their practical assignment were different from the sections used in the pre and posttest. Further analyses, therefore, were also performed using adapted test scores, which were the total test scores minus the scores obtained from the cross‐sectional anatomy questions.

For all the inductive analyses, variables and outcomes of the statistical assessment were represented as mean with ± standard deviation (±SD) if normally distributed, or as a median with range (minimum–maximum) if not normally distributed. Statistical significance was assumed when *P* < 0.05. Cohen's* d* was used to calculate the effect size of the results. Linear regression analysis was performed to model the relationship between the test scores and other variables. Post hoc sample size analysis on the acquired data was conducted at a level of power of 80%, at a *P*‐value of 0.05, resulting in a C_p,power_ of 7.8.

### Assessment of Qualitative Data

The audio recording of the focus group interview was transcribed verbatim and analyzed using direct content analysis by two researchers (D.H. and L.v.d.H.) independently. The coding process was performed using Atlas.ti software, version 8.2.29.0 (Atlas.ti Scientific Software Development GmbH, Berlin, Germany). To develop a codebook organized into categories and themes, the two researchers met periodically to discuss findings and discrepancies. The deductive process, discrepancies, and interpretations were regularly discussed by the research team. The codes were grouped into families in order to recognize themes that were discussed during the focus group interview.

### Ethical Approval

This study was carried out in agreement with the Statement on the Declaration of Helsinki and the Ethical Conduct of Clinical Studies and was approved by The Netherlands Association for Medical Education (NVMO) and registered (NERB dossier number 974).

## RESULTS

### Descriptive Statistics and Mental Rotation Test Scores

No significant changes were found between the participants’ baseline characteristics in the GreyMapp‐AR and the control group. Baseline characteristics per group are presented in Table 1.

**Table 1:** Baseline Characteristics of the Studied Population

Group	Age in years Mean (±SD)	Males/Females (%)	Medicine/Biomedical Sciences (%)	MRT‐score Mean points (±SD)	Test‐Scores Mean % (±SD)		Adapted Test‐scores Mean % (±SD)^a^		Cognitive load scores^b^ after PA 1 Mean points (±SD)				Cognitive load scores after PA 2 Mean points (±SD)				IMMS^c^ Mean points (±SD)			
					Pre‐	Post‐	Pre‐	Post‐	I	E	G	T	I	E	G	T	A	R	C	S
Overall (*n* = 31)	19.2 (±1.7)	61.3/38.7	74.2/25.8	13.9 (±3.7)	22.6 (±6.9)	46.1 (±14.3)	35.5 (±10.7)	55.5 (±12.4)	3.0 (±0.9)	2.5 (±1.0)	1.7 (±1.0)	7.2 (±2.4)	3.5 (±1.1)	3.7 (±1.1)	2.9 (±1.4)	10.1 (±2.7)	3.6 (±0.6)	3.1 (±0.6)	3.2 (±0.6)	3.3 (±0.7)
GreyMapp‐AR Group (*n* = 15)	19.3 (±2.3)	60.0/40.0	86.7/13.3	14.1 (±2.7)	21.1 (±5.8)	37.3 (±8.4)	32.3 (±9.8)	50.0 (±10.2)	3.2 (±1.0)	2.5 (±1.0)	1.7 (±1.1)	7.3 (±2.6)	3.6 (±0.9)	3.1 (±1.0)	2.3 (±1.2)	11.3 (±2.7)	3.8 (±0.5)	3.2 (±0.6)	3.2 (±0.7)	3.4 (±0.7)
Control Group (*n* = 16)	19.1 (±0.8)	62.5/37.5	62.5/37.5	13.6 (±4.5)	23.9 (±7.7)	54.3 (±14.0)	38.4 (±10.9)	60.6 (±12.4)	2.9 (±0.9)	2.4 (±1.0)	1.8 (±0.9)	7.1 (±2.3)	3.4 (±1.3)	4.3 (±1.0)	3.6 (±1.4)	7.3 (±2.3)	3.5 (±0.6)	3.1 (±0.6)	3.2 (±0.4)	3.2 (±0.7)

a Test scores after removal of the cross‐sectional questions;

b Cognitive load scores: I = intrinsic cognitive load score; E = extraneous cognitive load score; G = germane cognitive load score; T = total cognitive load score; IMMS, Instructional Measure of Motivation Survey; A = Attention; C = Confidence; R = Relevance; S = Satisfaction;

c Analyses were carried out in a smaller sample size (*n* = 14) due to premature termination of the experiment by one of the participants within the GreyMapp‐group; MRT, Mental Rotation Test; PA, practical assignment.

### Pre and Posttest Results

No significant differences were found between the pretest scores of the GreyMapp‐AR or the control group. Although both groups of participants showed significant improvement on the posttest scores with regard to the pretest scores, the posttest scores increased significantly more in the control group than in the GreyMapp‐AR group. When analyzing the three parts of the test separately, it was found that students from the control group scored significantly better on the cross‐sectional anatomy questions than the students in the GreyMapp‐AR group. The other two tests were not significantly different. This significant difference between both groups on the posttest could be reversed by excluding the cross‐sectional anatomy questions and by correcting for the pretest scores. Total pre and posttest scores were found to have an internal validity of *α* = 0.620. Table 1 presents an overview of the mean test scores.

Repeated Measures ANCOVA showed a significant effect of the factor Time, *F*_(1.3)_ = 128.7, *P* < 0.01 and of the factor Group, *F*_(1.3)_ = 12.9, *P* < 0.01. This indicates that both groups showed a significant improvement over time, although the control group showed a larger increase on test scores (*F*_(1.3)_ = 11.9, *P* < 0.01). This resulted in a mean adapted pretest score of 32.3 ± 9.8% and 38.4 ± 10.9% and adapted posttest score of 50.0 ± 10.2 and 60.6 ± 12.4% for the GreyMapp‐AR and the control group, respectively.

Adapted pre and posttest scores were found to have an internal validity of *α* = 0.547. The difference of the adapted pre‐test scores between the GreyMapp‐AR group and the control group showed not to be statistically significant (*P* = 0.113; Cohen's *d* = −0.59). The difference as measured by the adapted posttest scores between groups, however, showed to be statistically significant (*P* = 0.014; Cohen's *d* = 0.93). After correction for the pretest scores, no significant difference was found between groups (*P* = 0.359).

Repeated measures ANCOVA showed there was a significant effect of the factor Time, *F*_(1.3)_ = 67.4, *P* < 0.01 and of the factor Group, *F*_(1.3)_ = 7.4, *P* = < 0.05 in the adapted test setting, indicating that both groups showed a significant improvement over time. There was no interaction effect, *F*_(1.29)_ = 0.87, *P* = n.s., reaffirming that the learning improvement was the same for both the groups when taking the adapted test scores into account. Linear regression analysis showed that the learning method (GreyMapp‐AR vs. cross‐sections) and the pre and posttest scores were correlated significantly (*P* = 0.035; *R* = 0.380 and *P* < 0.01; *R* = 0.601).

### Cognitive Load Results

As expected, no difference in cognitive load was measured after the first practical assignment, although cognitive load increased significantly after the second practical assignment. The cognitive load after the second practical assignment, however, was exerted significantly more in the control group than in the GreyMapp‐AR group. In total, mean cognitive load scores after the first practical assignment and the second practical assignment were 7.2 ± 2.4 points and 10.1 ± 2.7 points, respectively, indicating a significant increase of cognitive load for all participants (*P* = 0.039; Cohen's *d* = −1.17). More details on the cognitive load scores can be found in Table 1 . Cognitive load after the first and second practical assignments showed not to be correlated with posttest scores (Tau‐b = 0.604, *P* = 0.070; Tau‐b = 0.135, *P* = 0.317) or adapted posttest scores (Tau‐b = 0.027, *P* = 0.848; Tau‐b = 0.053, *P* = 0.702).

### Instructional Measures of Motivation Survey Results

No significant differences were found in the subdomains of the IMMS between the two groups (Table 1). One participant of the AR group did not complete the IMMS questionnaire due to premature departure, resulting in the total number of participants being 30, with 14 in the GreyMapp‐AR group.

### Post hoc Sample Size Analysis

Post hoc sample size analysis showed that, with these data for pre and posttest scores, the investigated cohort should have consisted of seven students in each group, suggesting that a sufficient number of participants were included in this study.

### Students’ Opinions about the Effects of GreyMapp‐Augmented Reality Application and on Utilization of Cross‐sections in Neuroanatomy Learning

A total of eight participants contributed to the focus group interview (four males and four females; mean age 19.3 ± 1.4 years). Five participants worked with GreyMapp‐AR, and the remaining three students worked with the transverse sections. All participants were medical Bachelor’s students. Three themes were recognized after coding the transcribed focus group interview: (1) technological advances like GreyMapp‐AR in anatomy education; (2) advantages and disadvantages of GreyMapp‐AR and cross‐sections; and (3) opportunities and threats of GreyMapp‐AR.

#### Technological Advances in Anatomy Education

Technological advances as a whole in anatomy education were discussed during the focus group interview. Participants expressed little or no interest in technological advances, and some even said they missed old fashioned learning methods. Participants speculated that part of this problem was caused by faculty members’ incapability of working with innovative technologies, causing irritation by some participants. GreyMapp‐AR, however, was intuitive to use and provided an immediate and clear overview of the structures that needed to be learned. This enhanced fast learning of important structures, but also caused loss of detail and complexity. Participants who worked with GreyMapp‐AR expressed they missed details that were later seen in the cross‐sectional questions of the test. For example, the interviewees mentioned that the cortical surface of the brain was not highlighted in GreyMapp and was therefore not observed, although the white matter/grey matter demarcation and cortical gyrification pattern was clearly visible on the cross‐sections in the pre and posttest. Participants from the control group, however, explained that they already observed this gyrus‐sulcus pattern on the cross‐section which were used to study neuroanatomy during the second practical assignment. They could use this pattern as an additional anatomical landmark on which they based their recognition of subcortical structures. The participants stated that technological advances like GreyMapp‐AR could be useful, but that the learning objective should be more aligned with the learning method. For example, the details of the cross‐sections were not represented by GreyMapp‐AR, causing user frustration. The participants in the control group, however, complained about the difficulty of the questions testing 3D insights. Participants who worked with GreyMapp‐AR expressed that it was specifically well‐designed for learning 3D insights. The participants concluded that technological advances like GreyMapp‐AR could be used as additional learning tools but should never replace other teaching methods such as atlases, sections and the use of prosected specimens.

“I was a little disappointed and frustrated that I wasn’t enrolled in the GreyMapp‐AR group. It looked cool on the poster, and working with the preparation application [ed. CarMapp] was quite nice!” [S4]

“GreyMapp‐AR used these nice and bright colors which clearly defined certain structures. This was easy on the one hand, but a little too simplistic on the other. This became really frustrating for me when I saw the cross‐section questions in the second exam because I didn’t encounter this view during my assignments in the dissection room. [S1]”

#### Advantages and Disadvantages on Working with GreyMapp‐Augmented Reality Application and with Cross‐sections

With regard to the design of GreyMapp‐AR, it was very insightful that a structure was highlighted and named when it was pressed. Participants also stated, however, that they needed to get used to working with such a device. At the beginning, students did not like working with the marker as they needed to point the tablet camera toward this marker while holding the device with two hands, which complicated the learning process, according to the participants, as they could not take notes while holding the device and as holding it for a longer period of time also forced them into an uncomfortable position. Furthermore, the tablet screen was rather small, whereas the cross‐section could be seen from all angles. This also complicated learning, according to the participants.

The functionality of GreyMapp‐AR as an application to study neuroanatomy was confirmed. Participants expressed their concern about neuroanatomy learning because the internal structure of the brain is complex and lacks clear boundaries separating certain structures from others. In other anatomical regions, this differentiation is thought to be easier. However, participants of the GreyMapp‐AR group expressed that real human anatomy can only be observed in the dissection rooms and that GreyMapp‐AR should be used, therefore, to prepare for studying these structures in the dissection rooms.

“I held the tablet for quite some time and my arms got tired, so eventually I wanted to put the tablet down. But when I did so, the camera didn’t pick up the marker anymore, which led to the disappearance of the brain model in GreyMapp‐AR. [S6]”

“GreyMapp‐AR provided a very clear overview of the structures that we needed to identify, I believe. This clear overview could be very useful, especially when starting a neuroanatomy course! [S8]”

Participants expressed that working with cross‐sections was difficult but had some advantages as well. By requiring their maximum focus, students learned the 3D properties of the structures as they needed to build their own 3D model in their heads, based on the cross‐sections. However, participants also stated that, because of the complexity of the brain, learning with cross‐sections required more time.

“I worked really hard to recognize the structures at different levels in the different sections. This repetition of learning enabled me to remember anatomy for a longer period of time. [S3]”

#### Opportunities and Perceived Threats for GreyMapp‐Augmented Reality Application

Combining cross‐sections with GreyMapp‐AR would be the most optimal solution, according to the participants, although not necessarily at the same time. Participants expressed their concern regarding the short time they spent in the dissection rooms and wanted to use this time fully to study true human anatomy. All participants explicitly stated that they wanted to use GreyMapp‐AR to prepare for anatomy study but not to replace the use of specimens. As GreyMapp‐AR was considered to be more engaging than anatomy atlases, they expected, therefore, that future students would be more enthusiastic when they started to study neuroanatomy in the dissection rooms. They also expected that future students would be better prepared as GreyMapp‐AR provides a more comprehensive overview of the structures that need to be studied. When asked, participants expressed that they would appreciate the anytime‐anywhere principle. This indicated that they would like to prepare with GreyMapp‐AR at home for practical assignments in the dissection room.

“Such applications should never replace the real deal! I study medicine because I want to study the human body, not a computer. [S6]”

“I believe that such an application has some value, but more to prepare for the dissection room assignments. And I would like to be able to use it on your own tablet or smartphone at home. [S7]”

## DISCUSSION

### Primary Outcomes in Light of the Literature

#### Subscales of the Pre and Post‐test

This study showed that cross‐sectional neuroanatomy can best be studied using cross‐sections. Spatial knowledge can be studied equally effectively by either AR‐features such as GreyMapp‐AR or using cross‐sections. In addition, students in the GreyMapp‐AR group, experienced significantly less cognitive load, although the relevance of this outcome cannot be verified from the present results. These results are in line with the results from a meta‐analysis that demonstrated that 3D visualization techniques can be a potential and valuable addition to anatomy education (Yammine and Violato, 2015). Another meta‐analysis concluded that students can train spatial ability using cross‐sections and mental rotation exercises (Langlois et al., 2017). Other studies reaffirmed that cross‐sectional anatomy contributed significantly to visualizing anatomical structures in three dimensions (Lord, 1985; Provo et al., 2002; Samarakoon et al., 2016) underlining that studying neuroanatomy using cross‐sections significantly improves cross‐sectional anatomy knowledge.

To explain current results, it is hypothesized that the study materials in the control group (i.e., studying cross‐sections) were more in line with the examination (i.e., questions regarding cross‐sectional anatomy), which may have affected the outcomes of this study (Biggs, 1996; Dames, 2012). In the literature on assessment, this is known as the effect of constructive alignment of learning task and assessment method. The principle of constructive alignment is that the teaching activities, tasks, and assessments address the intended learning outcomes (Biggs, 1996; Dames, 2012).

Training with materials which are also included in a test provides students with the opportunity to construct several types of mnemonic devices (Bellezza, 1981) which are known to enhance memory on structural and functional levels (Karpicke and Smith, 2012; Rowland and DeLosh, 2015; Dresler et al., 2017; Lewis et al., 2018). Students who worked with transverse sections of the human brain, for example, observed certain landmarks that could be helpful in recognizing the same structures in sagittal or coronal sections of the human brain. This landmark point‐based registration (i.e., affine registration) is an important tool in medical image analysis and other applications in medicine (Haker et al., 2004; Papademetris et al., 2004). One of the exemplary landmarks, the striatum, is often recognized due to its striated appearance when transected in either one of the three planes. Such landmarks could not be observed using GreyMapp‐AR. The fact that students in the GreyMapp‐AR group missed such mnemonic devices could explain the significant differences in posttest scores of the questions that pertained to cross‐sections.

Transfer of learning, moreover, may have influenced this experiment, as learning in one context (i.e., the control group working with cross‐sections in practical assignment 2) could have enhanced a related performance in another context (i.e., the cross‐sectional test) (Perkins and Salomon, 1994). Hence, the presented results and possible explanations for the observed effect suggest that cross‐sectional anatomy can best be studied using cross‐sections. Teaching and studying cross‐sectional anatomy, although demanding, remains of crucial importance for students. Current doctors routinely encounter cross‐sectional anatomy, and with the rapid development of modern medical imaging technologies, the number of future doctors who will be working with cross‐sectional anatomy will increase as well (Samarakoon et al., 2016). It remains of crucial importance, therefore, for (bio)medical students to study cross‐sectional anatomy.

In order to improve the GreyMapp application, the source data (MR slices in the three anatomical directions) could be added to the 3D models in the AR environment. A similar feature interleaving sectional and whole neuroanatomy has been described by Pani et al. (2013). This feature was found to be more efficient than a basic transfer paradigm (i.e., whole neuroanatomy first and sectional neuroanatomy later) and neuroanatomy learning using cross‐sections only (Pani et al., 2013).

Such outcomes have also been described in the recent study by Moro et al. (2017), in which the investigators compared different computer‐based learning techniques (i.e., tablet‐based AR, AR, and virtual reality) and their impact on gaining knowledge of anatomy. They found that these three methods did not differ significantly with regard to gaining knowledge of anatomy, although it was found that these new methods promote intrinsic benefits such as increased learner immersion and engagement (Moro et al., 2017).

### Secondary Outcomes in Light of the Literature

Participants in the cross‐section group experienced a significantly higher extraneous and germane cognitive load. Extraneous cognitive load is related to the quality of the instruction design; germane cognitive load is related to processes that contribute to the construction and automation of schemas (Paas and Van Merriënboer, 1994). This suggests that it is difficult for students to study neuroanatomy using cross‐sections, a trend that has been observed by others as well (Brunken et al., 2003; Anglin et al., 2004; Wilson, 2015). This might indicate that cross‐sections are not appropriate learning materials for novice learners. Sections could be introduced into anatomy education at later stages to teach students cross‐sectional anatomy of body parts.

The significant differences found in germane and extraneous cognitive load, however, did not appear to interfere with the test results. Küçük et al. (2016) also stated that students who worked with AR applications reported to experience less cognitive load than students who studied anatomy using a textbook (Küçük et al., 2016). Other studies also found that, when AR applications are well designed, less cognitive effort can be achieved when using these applications for the study of anatomy (Iordache et al., 2012; Di Serio et al., 2013).

Based on the literature, it was expected that GreyMapp‐AR would improve the attractiveness and effectiveness of learning processes (Lee, 2012), thus increasing students’ motivation to study neuroanatomy (Shen et al., 2008; Huang et al., 2010). Quantitative measurements of the different components of the IMMS, however, showed no significant differences between the two groups. This would indicate that the participants in either group were not more or less motivated to study neuroanatomy although qualitative results show that the participants that did not work with GreyMapp‐AR were disappointed. A possible explanation for these incongruent findings could be that all students were enthusiastic about the practical assignments and materials to work with as they had never encountered neuroanatomical tissues and had never studied neuroanatomy prior to this experiment.

### Strengths and Limitations of the Study

One of the strengths of this study was that it investigated classic anatomy education by comparing cross‐sections of specimens with an AR application as a superiority/inferiority study instead of applying an add‐on protocol. Another strength of this study lies in the questions that made up the pre and posttests. All questions assessed 3D knowledge of anatomy, thus indicating the complexity of the domain of 3D anatomy.

One limitation of this study was the internal validity and reliability of the two tests, which was found to be questionable according to the performed Cronbach’s alpha assessment. Another limitation was that it was carried out in one evening, with the two practical assignments taking only 20 minutes per practical assignment. Another possible limitation of this study concerns the qualitative assessment. The eight‐week period between the experiment and the focus group interview, firstly, might have caused students to forget subtle details of the experiment, the setting, the cross‐sections and the design of GreyMapp‐AR. Although this was not noted during the focus group interview, it cannot be ruled out that participants might have had trouble remembering fine details. The fact that the subgroup that took part in the qualitative assessment was a non‐representative section of the total group of students who participated in the experiment itself, secondly, is another limitation. This preludes drawing sound conclusions with regard to the qualitative data. In future research, it is recommended to implement AR for a longer period, for a few days or an entire course, for instance, to investigate the results of using AR over a longer time span. When the time span of the experiment is extended, however, it may be harder to control what students study outside the experimental setting. Another limitation of this study is the inclusion of cross‐sectional imaging in the control group and testing on cross‐sectional anatomy. To correct for this, a subanalysis was run from which the cross‐sectional anatomy questions were excluded. In order to minimize the effect of this limitation on the discussion and conclusions that can be drawn from this study, only the adapted test results were discussed and used to draw conclusions and provide recommendations. Despite the post hoc sample size analysis, which showed that sufficient participants were included in each group, the limited number of included participants is a possible limitation of this study. Though this limited number of included participants is in line with other, similar studies (Birt et al., 2018), larger sample sizes have also been described (Drapkin et al., 2015; Küçük et al., 2016). Future studies should aim to include a larger sample size, if possible, as larger pooled sample sizes are likely to be more accurate and to reflect true population statistics and associations more closely (Yammine, 2014).

## CONCLUSIONS

The present study shows that students learning 3D anatomy of deep structures of the brain using cross‐sections only outperform students using GreyMapp‐AR on those test questions that are based on cross‐sections. When questions regarding cross‐sectional anatomy were excluded, no significant difference between the learning effects of the GreyMapp‐AR and control group were observed. Nevertheless, students experienced less cognitive load when working with GreyMapp‐AR and consider GreyMapp‐AR to be a valuable addition to more traditional learning methods. The effectiveness of GreyMapp and other AR applications could be further improved by adding a cross‐sectional feature in which the source data (i.e., MRI slices) could be added to the application in order to provide students with the best of both worlds. These recommendations, however, should be interpreted with care due to limitations in the study design.

## NOTES ON CONTRIBUTORS

DYLAN J.H.A. HENSSEN, M.D., is a graduate (Ph.D.) student in the Departments of Anatomy and Neurosurgery at the Radboud University Medical Center, Nijmegen, The Netherlands. He teaches gross anatomy, embryology, and focuses on neuroanatomy education in a variety of courses. He is also a member of several educational boards and committees and his research interest is in using innovative (pre)clinical imaging and post‐processing methods to assess brain structure and function

LOES VAN DEN HEUVEL, M.Sc., is a master’s degree student in (Linguistics) in the Department of Educational Sciences, Faculty of Social Sciences, Radboud University, Nijmegen, The Netherlands. She has a master’s degree in Educational Sciences from Radboud University and portions of this study design are included in her master’s degree dissertation.

GUIDO DE JONG, M.Sc., is a graduate (Ph.D.) student in the Department of Neurosurgery at the Radboud University Medical Center, Nijmegen, The Netherlands. He has a master’s degree in Technical Medicine.

MARC A.T.M. VORSTENBOSCH, Ph.D., is an associate professor and principal lecturer in the Department of Anatomy at the Radboud University Nijmegen Medical Centre, Nijmegen, The Netherlands. He teaches gross anatomy, functional anatomy of the locomotor system, and head and neck anatomy. As a researcher, he is involved in several research projects on anatomy education and assessment of medical competence.

ANNE‐MARIE VAN CAPPELLEN VAN WALSUM, M.D., Ph.D., is an assistant professor and junior principal lecturer in the Department of Anatomy at the Radboud University Nijmegen Medical Centre, Nijmegen, The Netherlands. She teaches anatomy and neuroanatomy to medical students and Technical Medicine students. Her research interest is in studying brain networks in both pathological and non‐pathological tissues.

MARIANNE M. VAN DEN HURK, Ph.D., is an assistant professor in Educational Psychology at the Faculty of Social Sciences, Radboud University Nijmegen, Nijmegen, The Netherlands. She teaches courses on educational sciences, learning, and instruction and professional learning. Her research interest is in the area of learning processes taking place in instructional environments that promote active learning in higher education.

JAN G.M. KOOLOOS, Ph.D., is an associate professor and senior principal lecturer in the Department of Anatomy at the Radboud University Nijmegen Medical Centre, Nijmegen, The Netherlands. He teaches gross anatomy, embryology, histology, and functional anatomy of the locomotor system in a variety of curricula. He is also Co‐President of Faculty Development and involved in several research projects on anatomy education.

RONALD H.M.A. BARTELS, M.D. Ph.D., is a professor and chair of the Department of Neurosurgery at Radboud University Nijmegen Medical Centre, Nijmegen, The Netherlands. He is a neurosurgeon and supervises several research projects on teaching clinically relevant neuroanatomy.
