# Supplementary figures and images for: Neuroanatomy Learning: Augmented Reality vs. Cross‐Sections
Source: Anat Sci Educ. 2019 Jul 19;13(3):353–65. doi: 10.1002/ase.1912 (PMC7317366; doi:10.1002/ase.1912)

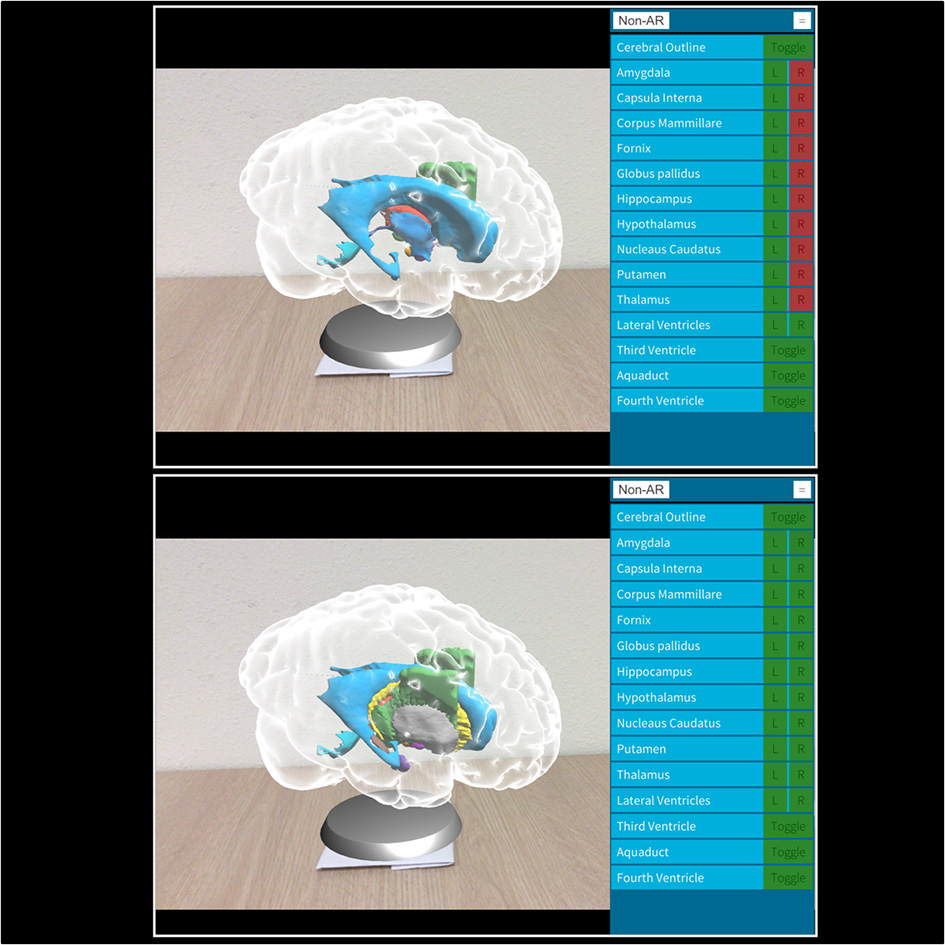

Supplement: Supplementary file 1 [file ASE-13-353-s001.tif]
