# Supplementary material for: Neuroanatomy Learning: Augmented Reality vs. Cross‐Sections
Source: Anat Sci Educ. 2019 Jul 19;13(3):353–65. doi: 10.1002/ase.1912 (PMC7317366; doi:10.1002/ase.1912)
Supplement: Supplementary file 2 [file ASE-13-353-s002.docx]

SUPPLEMENTARY FILE 1

**Part 1: Extended-matching questions**

**Optional answers:**

1. amygdala
2. aqueduct
3. internal capsule
4. third ventricle
5. fornix
6. globus pallidus
7. hippocampus
8. hypothalamus
9. lateral ventricle
10. caudate nucleus
11. putamen
12. thalamus
13. fourth ventricle

## Instruction:

## Choose the most correct option(s) from the aforementioned list of structures to answer the separate questions. The number in parentheses indicates the maximum number of points and the maximum number of answers that can be given.

| 1 | Which structure can be found in between the caudate nucleus and the fornis? | (1) | Lateral ventricle |  |  |
| --- | --- | --- | --- | --- | --- |
| 2 | Which structures lie adjacent to the third ventricle? | (2) | Thalamus | Hypothalamus |  |
| 3 | Directly adjacent to the medial side of the internal capsula, we find the…. ? | (2) | Caudate nucleus | Thalamus | Hypothalamus |
| 4 | Which structures are found directly medial and superior to the lateral/temporal horn of the lateral ventricles? | (2) | Hippocampus | Caudate nucleus |  |
| 5 | Which structures posses the same curved shape as the caudate nucleus? | (2) | Fornix | Lateral ventricle |  |

**Part 2: Multiple choice questions**

| In relation to the head of the caudate nucleus, the internal capsula is positioned:   1. more lateral 2. more medial | In relation to the internal capsule, the thalamus is found:   1. more lateral 2. more medial |
| --- | --- |
| In relation to the putamen, the internal capsule is found:   1. more lateral 2. more medial | In relation to the internal capsule, the globus pallidus is found:   1. more lateral 2. more medial |
| In relation to the thalamus, the amygdala has a more…. position:   1. more lateral 2. more medial | In relation to the hypothalamus, the thalamus is found:   1. inferior 2. superior |
| In relation to the amygdala, the head of the caudate nucleus is found:   1. more dorsal 2. more ventral | In relation to the thalamus, the head of the caudate nucleus is found:   1. more dorsal 2. more ventral |
| In relation to the lateral ventricle, the fornix is found:   1. more lateral 2. more medial | The hippocampus is found …. to the fornix:   1. lateral 2. medial |
| In relation to the hippocampus, the temporal horn of the lateral ventricle is found:   1. more lateral 2. more medial |  |

| 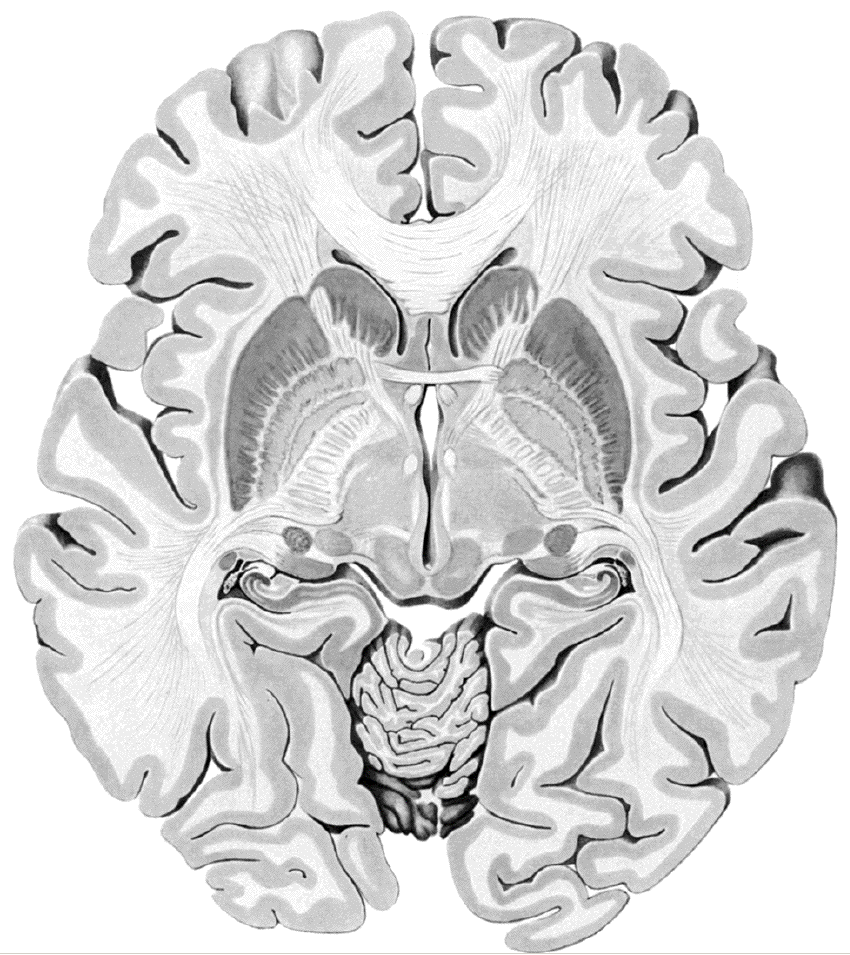  **D**  **Part 3: Cross-sectional anatomy**  **A**  **B**  **E**  **C** |
| --- |
| This figure shows a transversal section of the brain. Five structures are encircled and annotated A-E. Provide the correct anatomical terms for structures A-E below:   \| A \| Fornix \| \| --- \| --- \| \| B \| Tail of the caudate nucleus \| \| C \| Globus pallidus \| \| D \| Hippocampus \| \| E \| Internal capsule \| |

#

| 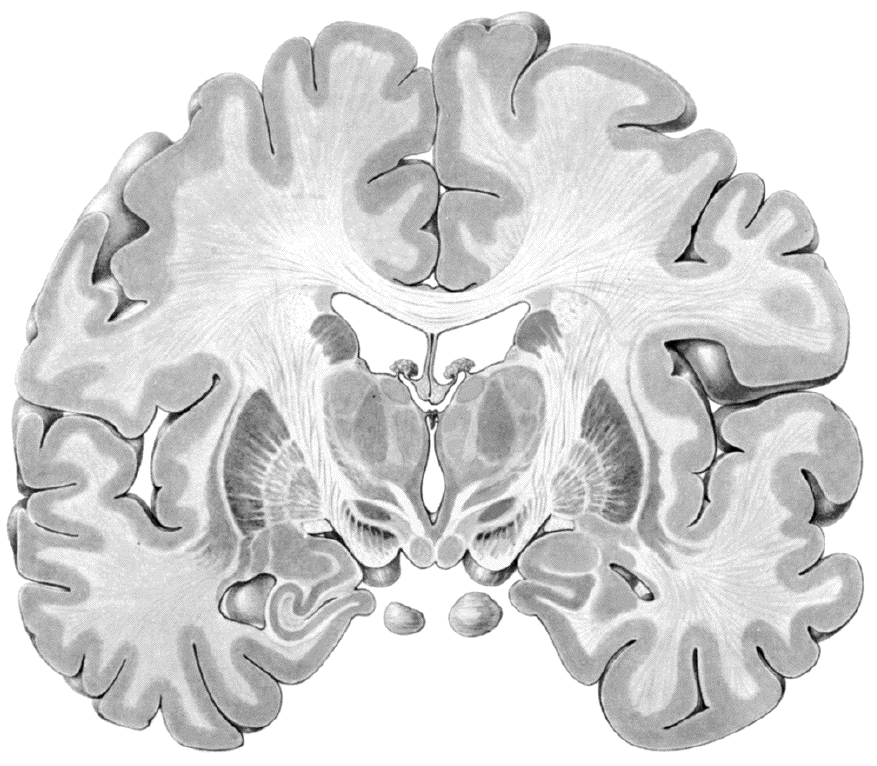  **A**  **C**  **E**  **B**  **D** |
| --- |
| This figure shows a frontal section of the brain. Five structures are encircled and annotated A-E. Provide the correct anatomical terms for structures A-E below:   \| A \| Hypothalamus \| \| --- \| --- \| \| B \| Lateral ventricle \| \| C \| Hippocampus \| \| D \| Internal capsule \| \| E \| Globus pallidus \| |

| 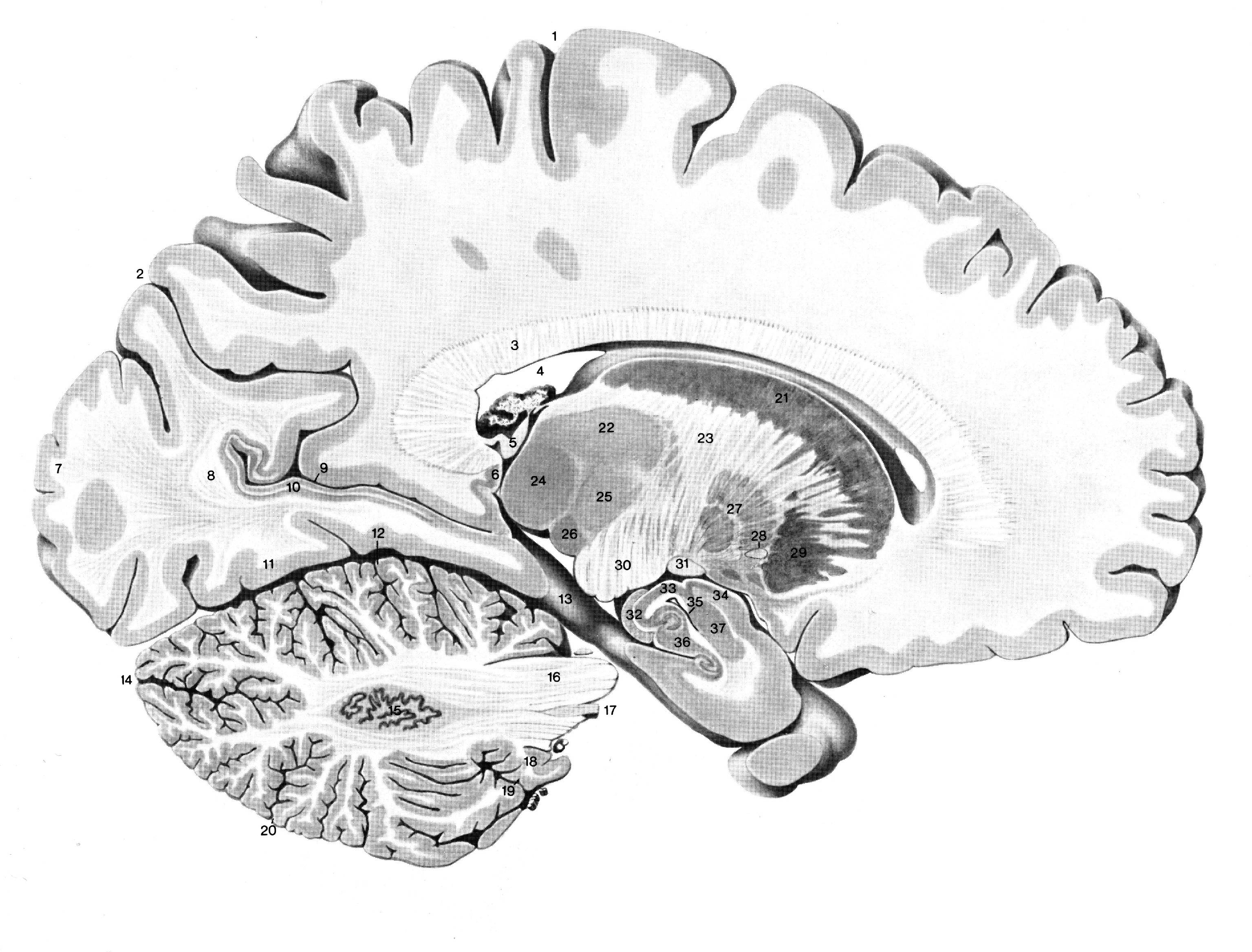  **D**  **E**  **C**  **B**  **A** |
| --- |
| Bovenstaande afbeelding is een saggitale doorsnede van het brein. Vijf structuren zijn omcirkeld en genummerd A t/m E. Geef in onderstaande tabel de namen van de structuren zo nauwkeurig mogelijk weer.   \| A \| Caudate nucleus \| \| --- \| --- \| \| B \| Globus pallidus \| \| C \| Putamen \| \| D \| Hippocampus \| \| E \| Lateral ventricle \| |
