# Supplementary material for: Neuroanatomy Learning: Augmented Reality vs. Cross‐Sections
Source: Anat Sci Educ. 2019 Jul 19;13(3):353–65. doi: 10.1002/ase.1912 (PMC7317366; doi:10.1002/ase.1912)
Supplement: Supplementary file 3 [file ASE-13-353-s003.docx]

SUPPLEMENTARY FILE 2

**Cognitive Load Questions** (Cierniak et al., 2009)

*[1] How difficult was the learning content for you?*

1. Not at all

2. Just a little bit

3. Somewhat

4. Pretty much

5. Very

6. Extremely

*[2] How difficult was it for you to learn with the material?*

1. Not at all

2. Just a little bit

3. Somewhat

4. Pretty much

5. Very

6. Extremely

*[3] How much did you concentrate during learning?*

1. Not at all

2. Just a little bit

3. Somewhat

4. Pretty much

5. Very

6. Extremely
