# Supplementary material for: Neuroanatomy Learning: Augmented Reality vs. Cross‐Sections
Source: Anat Sci Educ. 2019 Jul 19;13(3):353–65. doi: 10.1002/ase.1912 (PMC7317366; doi:10.1002/ase.1912)
Supplement: Supplementary file 4 [file ASE-13-353-s004.docx]

SUPPLEMENTARY FILE 3

**Instructional Materials Motivation Survey**

(Keller, 2010, pp. 283—284)

Instructions

*Instructional Materials Motivation Survey*

There are 36 statements in this questionnaire. Please think about each statement in relation to the instructional materials you have just studied and indicate how true it is. Give the answer that truly applies to you, and not what you would like to be true, or what you think others want to hear.

Think about each statement by itself and indicate how true it is. Do not be influenced by your answers to other statements.

Record your responses on the answer sheet that is provided and follow any additional instructions that may be provided in regard to the answer sheet that is being used with this survey. Thank you.

Use the following values to indicate your response to each item.

1 (or A) = Not true

2 (or B) = Slightly true

3 (or C) = Moderately true

4 (or D) = Mostly true

5 (or E) = Very true

The survey contains four parts: (1) attention (12 questions); (2) relevance (9 questions); (3) confidence (9 questions) and; (4) satisfaction (6 questions). The parts are not viewed separately, but the questions are mixed throughout the survey (maximum score: 180 points; 5 points for each question)

01C01 When I first looked at this lesson, I had the impression that it would be easy for me.

02A01 There was something interesting at the beginning of this lesson that got my attention.

03C02 This material was more difficult to understand than I would like for it to be.*

04C03 After reading the introductory information, I felt confident that I knew what I was

supposed to learn from this lesson.

05S01 Completing the exercises in this lesson gave me a satisfying feeling of

accomplishment.

06R01 It is clear to me how the content of this material is related to things I already know.

07C04 Many of the pages had so much information that it was hard to pick out and remember

the important points.*

08A02 These materials are eye-catching.

09R02 There were stories, pictures, or examples that showed me how this material could be

important to some people.

10R03 Completing this lesson successfully was important to me.

11A03 The quality of the writing helped to hold my attention.

12A04 This lesson is so abstract that it was hard to keep my attention on it.*

13C05 As I worked on this lesson, I was confident that I could learn the content.

14S02 I enjoyed this lesson so much that I would like to know more about this topic.

15A05 The pages of this lesson look dry and unappealing.*

16R04 The content of this material is relevant to my interests.

17A06 The way the information is arranged on the pages helped keep my attention.

18R05 There are explanations or examples of how people use the knowledge in this lesson.

19C06 The exercises in this lesson were too difficult.*

20A07 This lesson has things that stimulated my curiosity.

21S03 I really enjoyed studying this lesson.

22A08 The amount of repetition in this lesson caused me to get bored sometimes.*

23R06 The content and style of writing in this lesson convey the impression that its content is

worth knowing.

24A09 I learned some things that were surprising or unexpected.

25C07 After working on this lesson for a while, I was confident that I would be able to pass a

test on it.

26R07 This lesson was not relevant to my needs because I already knew most of it.*

27S04 The wording of feedback after the exercises, or of other comments in this lesson,

helped me feel rewarded for my effort.

28A10 The variety of reading passages, exercises, illustrations, etc., helped keep my attention

on the lesson.

29A11 The style of writing is boring.*

30R08 I could relate the content of this lesson to things I have seen, done, or thought about in

my own life.

31A12 There are so many words on each page that it is irritating.*

32S05 It felt good to successfully complete this lesson.

33R09 The content of this lesson will be useful to me.

34C08 I could not really understand quite a bit of the material in this lesson.*

35C09 The good organization of the content helped me be confident that I would learn this

material.

36S06 It was a pleasure to work on such a well-designed lesson.

* Asterisked items should be recoded prior to data analysis (1 = 5, 2 = 4, 4 = 2, and 5 = 1).

1 02A01 is the second item of the IMMS scale, and the first item of the A construct, 03C02 is the third item of the IMMS scale, and the second item of the C construct, etc. Codes were added for reference; in the original IMMS, items are numbered 1 through 36.
